# Better Perceived Senior Center Quality was associated with Less Subjective Cognitive Decline

**DOI:** 10.1093/geroni/igaf122.2047

**Published:** 2025-12-31

**Authors:** Hui “Jimmy” Xie, Xiao Zhang, Cinthia Camacho, Maryna Bankovska, Bing Han, Deborah Cohen

**Affiliations:** California State University, Northridge, Northridge, California, United States; California State University, Northridge, Northridge, California, United States; California State University, Northridge, Northridge, California, United States; California State University, Northridge, Northridge, California, United States; Kaiser Permanente Southern California, Pasadena, California, United States; Kaiser Permanente Southern California, Pasadena, California, United States

## Abstract

Senior centers offer various programs and activities that are beneficial to brain health such as exercise classes, arts and craft, and social activities. Despite that, there is a dearth of research on the role of senior center in older adults’ cognitive health. Using data collected at two time points, this study examined the association between perceived senior center quality (PSCQ) and older adults’ subjective cognitive decline (SCD). PSCQ was measured using an 8-item, 5-point scale adapted from Park Quality Scale, addressing senior center’s built/social environment and programming. SCD was measured using the 14-item Cognitive Function Instrument. Control variables included gender, race/ethnicity, age, marital status, education, senior center attendance frequency, and physical and mental health (measured using SF-12 questionnaire). Data were collected in 2023 (PSCQ, demographics, physical/mental health) and 2024 (SCD, attendance frequency) from 152 older adults at 6 senior centers in Los Angeles and neighboring counties. Linear mixed effects model was used to examine the relationship between PSCQ and SCD. Of all the participants (mean age = 74), 78% were female, 39% were married, and 44% had a college or higher degree. As for race/ethnicity, 33% were White, 31% were Asian and 26% were Hispanic. After controlling for demographics, attendance frequency, and physical/mental health, PSCQ was negatively associated with SCD (b=-.89, p<.05). The results highlight the importance of senior center quality to older adults’ cognitive health. Future studies should examine potential mechanisms underlying the relationship between PSCQ and cognitive health.

